# Cardiff Online Cognitive Assessment in a National Sample: Cross-Sectional Web-Based Study

**DOI:** 10.2196/46675

**Published:** 2023-09-13

**Authors:** Amy Joanne Lynham, Ian R Jones, James T R Walters

**Affiliations:** 1 Division of Psychological Medicine, School of Medicine, Cardiff University Cardiff United Kingdom

**Keywords:** cognition, digital assessment, mental health, mobile phone, normative data, web-based, cognitive assessment, CONCA

## Abstract

**Background:**

Psychiatric disorders are associated with cognitive impairment. We have developed a web-based, 9-task cognitive battery to measure the core domains affected in people with psychiatric disorders. To date, this assessment has been used to collect data on a clinical sample of participants with psychiatric disorders.

**Objective:**

The aims of this study were (1) to establish a briefer version of the battery (called the Cardiff Online Cognitive Assessment [CONCA]) that can give a valid measure of cognitive ability (“g”) and (2) to collect normative data and demonstrate CONCA’s application in a health population sample.

**Methods:**

Based on 6 criteria and data from our previous study, we selected 5 out of the original 9 tasks to include in CONCA. These included 3 core tasks that were sufficient to derive a measure of “g” and 2 optional tasks. Participants from a web-based national cohort study (HealthWise Wales) were invited to complete CONCA. Completion rates, sample characteristics, performance distributions, and associations between cognitive performance and demographic characteristics and mental health measures were examined.

**Results:**

A total of 3679 participants completed at least one CONCA task, of which 3135 completed all 3 core CONCA tasks. Performance on CONCA was associated with age (B=–0.05, SE 0.002; *P*<.001), device (tablet computer: B=–0.26, SE 0.05; *P*<.001; smartphone: B=–0.46, SE 0.05; *P*<.001), education (degree: B=1.68, SE 0.14; *P*<.001), depression symptoms (B=–0.04, SE 0.01; *P*<.001), and anxiety symptoms (B=–0.04, SE 0.01; *P*<.001).

**Conclusions:**

CONCA provides a valid measure of “g,” which can be derived using as few as 3 tasks that take no more than 15 minutes. Performance on CONCA showed associations with demographic characteristics in the expected direction and was associated with current depression and anxiety symptoms. The effect of device on cognitive performance is an important consideration for research using web-based assessments.

## Introduction

### Background

Cognitive function has been shown to be associated with health, such that those who perform better on cognitive assessments have better health outcomes, including decreased mortality risk, on average, than those with lower cognitive function [1-4]. A number of mental and physical conditions are associated with cognitive impairments, including common conditions such as depression [5], anxiety [6], hypertension [7] and diabetes [8]. More pronounced cognitive impairments are seen in those with a diagnosis of a severe mental disorder, such as schizophrenia [9] or bipolar disorder [10]. The severity of these impairments is an important predictor of occupational and social functioning in participants diagnosed with these disorders [11,12].

Existing cognitive research is limited by sample size, as collecting cognitive data traditionally involves a face-to-face assessment and can be labor-intensive. However, the rise in internet use over the past few decades and the development of digital assessments have presented researchers with new opportunities to collect large data sets [13]. At the Medical Research Council Centre for Neuropsychiatric Genetics and Genomics, we have developed and used a web-based cognitive assessment to collect data on over 1000 participants diagnosed with a range of psychiatric disorders [14]. To date, we have (1) established validity against a gold-standard measure of cognition in psychiatric research (Measurement and Treatment Research to Improve Cognition in Schizophrenia [MATRICS] Consensus Cognitive Battery [MCCB]); (2) reported an association between performance on the battery and functioning in a cohort of participants with psychiatric disorders; and (3) demonstrated that performance on the battery discriminates between controls and participants with schizophrenia, those with bipolar disorder, and those with major depressive disorder. However, we have not reported normative data for the battery, measured the distribution of scores, or examined associations between performance on the battery and demographic factors in a population sample. Although normative data for some of the individual tasks already exist, it is crucial that normative data for web-based tasks be collected on the web using the same platform [13]. In addition, the original battery consisted of 9 tasks with an administration time of up to 50 minutes. However, given that some of the correlations between the web-based tasks and the MCCB were small and there were concerns about the length of the battery, we have developed a briefer version of the battery with an improved user-friendly interface (Cardiff Online Cognitive Assessment [CONCA]). This new version of the battery was specifically designed to provide a brief, valid measure of general cognitive function (“g”). A measure of general cognition (“g”) was considered appropriate given the literature showing that cognitive impairment in psychiatric disorders (particularly schizophrenia) is characterized by widespread, global impairment rather than specific localized dysfunction, and this global impairment is predictive of poor community functioning [15].

### HealthWise Wales

In addition to cognitive assessments, web-based technologies have provided the opportunity to recruit population cohorts for epidemiological research. One such cohort is HealthWise Wales, a Welsh Government–funded digital health project that has recruited a web-based cohort of people living or receiving health care in Wales [16]. The aim of HealthWise Wales is to understand factors that impact health and well-being, including social inequalities, the environment, and health behaviors, through web-based data collection and linkage to routine health care records. This cohort provides an opportunity to examine cognitive performance in the general population.

### Study Aims

This study had 2 aims. First, we established a core battery (CONCA) that can provide a valid measure of “g” in less than 15 minutes. To do this, we used data from our previous study [14] to evaluate the original 9 cognitive tasks against set criteria. Second, we aimed to derive normative data for CONCA and demonstrate its application in a health population sample by collecting cognitive data from HealthWise Wales. This study is presented in 2 parts to reflect these aims.

## Methods

### Part 1: Establishing CONCA

#### Participants

Full details of the original study have been published previously [14]. Briefly, participants were recruited from the databases of 2 existing studies of psychiatric disorders within the Medical Research Council Centre for Neuropsychiatric Genetics and Genomics: Cognition in Mood, Psychosis, and Schizophrenia Study (CoMPaSS [17]) and the National Centre for Mental Health (NCMH [18]). For the purposes of this study, we included only participants with data on the full 9 tasks (N=841).

#### Measures

The CONCA was developed to assess cognitive function in individuals with a history of mental illness. All tasks (including source code) were developed by The Many Brains Project, a not-for-profit organization that develops open-source, web-based tools to assess cognitive function [19,20]. We selected 9 tasks to assess, as closely as possible, the domains outlined by the National Institute for Mental Health’s MATRICS initiative [21]. To improve the battery, we aimed to reduce the length to 5 tasks with a maximum administration time of 30 minutes that would provide a brief, valid measure of “g.”

We selected the MCCB as our comparison measure to validate CONCA due to the rigorous selection procedure used in its development and its widespread adoption in mental health research. The MCCB consists of 10 pen-and-paper tasks assessing the 7 domains outlined by the MATRICS initiative [21]. It was developed using expert panels, consultations with scientists, evaluations of psychometric properties, and assessments of tolerability and practicality, with the explicit aim of creating a gold-standard battery for use in schizophrenia research [22].

Participants also completed the 12-item version of the World Health Organization Disability Assessment Schedule (WHODAS [23]), which assesses 6 domains of functional impairment: understanding and communicating, mobility, self-care, social interactions, life activities, and participation in the community.

#### Study Design

The study design was cross-sectional. The selection of tasks for the new CONCA battery was guided by the findings in our previous study [14] and we additionally conducted some new analyses. This study design has been previously described [14] but briefly, participants who had consented to be contacted about follow-up studies were invited through email or letter to complete the original 9-task battery. A subset of participants (N=65) additionally completed the MCCB as a gold-standard comparison measure.

Following discussions within our research team and consultation with our health professionals and patient representatives, we outlined 6 criteria to be used to guide task selection. To be considered for inclusion, we sought to demonstrate that each task was (1) correlated with its equivalent task in the MCCB, (2) correlated with general cognitive function “g” derived using the MCCB, (3) associated with functioning as measured by the WHODAS [23], (4) loaded onto a measure of “g” derived from the 9-task battery using factor analysis, (5) considered acceptable based on participant feedback with no insurmountable technical issues reported, and (6) translatable into other languages to support our international collaborations. Tasks were considered “translatable” if it would be possible to translate the instructions and materials without fundamentally changing the measurement properties of the task (eg, tasks with nonverbal stimuli). Correlations between the CONCA tasks and the MCCB (criteria 1 and 2), associations with functional outcomes (criterion 3), and technical issues and participant feedback (criterion 5) have been previously published in Lynham et al [14] (a summary of these results can be found in Table S1 in Multimedia Appendix 1). We conducted further analyses (see Analysis section) to determine whether tasks met criterion 4 and to evaluate the validity of the new battery. As far as possible, we selected tasks that were representative of different domains as opposed to similar tasks to ensure CONCA was a well-rounded measure of global cognitive function.

#### Analysis

The structure of the 9-task web-based cognitive battery was examined using exploratory factor analysis. The number of factors was identified using scree plots and parallel analysis. Principal axis factoring with oblique rotation (direct oblimin) was conducted to identify the factors.

To evaluate the validity of “g” derived using the new CONCA battery, we examined correlations between “g” derived using the MCCB and “g” derived using the new CONCA battery. This analysis was conducted on a subset of participants with MCCB data available (n=65). “g” was derived using multidimensional scaling [24], which is an approach analogous to principal component analysis, with the first component extracted as “g.”

### Part 2: Assessing Cognition in HealthWise Wales

#### Participants

Participants were recruited from HealthWise Wales, a web-based national population cohort [16]. Adults aged 16 years and older who live or receive their health care in Wales are eligible for inclusion in HealthWise Wales. Participants consent to being contacted for follow-up data collection with new questionnaires added to the website and advertised through email invitations every 6 months. HealthWise Wales data are collected and stored in the Secure Access Portal and Protected HealthWise Wales Information Repository (SAPPHIRe), which is powered by the UK Secure e-Research Platform (UKSeRP) [25]. The CONCA was added as a module on the HealthWise Wales website in January 2020, and email invitations were sent to all participants in the cohort (N=29,492). Ethical approval for CONCA was granted by Cardiff University’s School of Medicine Research Ethics Committee (reference: 15/64).

#### Measures

Participants completed CONCA, the WHODAS, and the Hospital Anxiety and Depression Scale (HADS [26]), as well as providing basic demographic information (age, gender, education, and device used). The collected data were also linked with existing data from HealthWise Wales to determine whether participants had ever been diagnosed with or treated for a mental health problem [16].

#### Study Design

The study design was cross-sectional. Participants completed the study by either clicking on the link in their email invitation or clicking on the module on the HealthWise Wales home screen. This took participants to the CONCA web page, where they could read the information sheet, provide informed consent, and complete all the measures.

#### Analysis

All analyses were conducted using R (version 3.6.1; The R Foundation). For each task, *z* scores were derived using the mean and SD of the sample. The 2 measures of “g” were derived using multidimensional scaling [24]: (1) using the scores on the 3 core CONCA tasks only (Core “g”), and (2) using scores on the complete (Full “g”). These 2 measures of “g” were highly correlated (*r*=0.93).

Completion rates for each task were calculated. To examine predictors of completing the optional tasks, we performed a logistic regression to test the association between completion of at least one optional task and the following variables: cognitive performance on the core tasks (Core “g”), age, gender, education, device, and having ever received a diagnosis or treatment for a mental health problem.

We performed multiple linear regression to test the association between cognitive performance (“g”) and the following demographic variables in a single model: age, gender, education, and device. We repeated this analysis for each cognitive task. *P* values were corrected using the false discovery rate method.

As CONCA was developed as a tool for mental health research, we evaluated whether performance on CONCA was associated with the following two measures of mental health: (1) whether participants had ever been diagnosed with or treated for a mental health problem; and (2) scores on the HADS subscales for depression and anxiety. Each mental health variable (ever diagnosed, HADS depression, HADS anxiety) was entered as a predictor into separate linear regressions with “g” as the outcome and age, gender, education, and device as covariates.

### Statement of Ethical Approval

Ethical approval for HealthWise Wales was obtained from Wales Research Ethics Committee 3 (reference: 15/WA/0076). Ethical approval for CONCA was granted by Cardiff University’s School of Medicine Research Ethics Committee (reference: 15/64). All participants indicated their informed consent by selecting “yes” in response to the statement, “I agree to take part in this study and know that I am free to leave the study at any point” at the start of the study. No personal identifiers were collected as part of the study, as all data were linked to an ID number. Participants did not receive compensation for their time.

## Results

### Part 1: Establishing CONCA

#### Factor Loadings

Examination of the scree plot and parallel analysis indicated 2 factors with eigenvalues above 1. All the measures except vocabulary and the balloon analogue risk task loaded onto the first factor (Table 1). Only vocabulary had a high load on the second factor.

**Table 1:** Factor loadings of the web-based tasks.

Task	Factor 1	Factor 2
Matrix reasoning	0.56	0.29
Multiple object tracking	0.7	0.04
Balloon analogue risk task	0.18	0.27
Backward digit span	0.43	0.29
Verbal paired associates test	0.4	0.24
Digit symbol coding	0.81	–0.11
Morphed emotion identification	0.56	0.07
Vocabulary	–0.07	0.66
Hartshorne visual working memory	0.66	–0.16
Proportion of variance explained by all tasks	0.76	0.24

#### Selection of the Final CONCA Battery

The final battery consisted of 3 core tasks with an administration time of 15 minutes and 2 optional tasks (total administration time of 30 minutes). Once the final tasks were selected, we consulted with patient representatives to design a new user-friendly website for CONCA [27].

#### Task 1: Digit Symbol Coding

This task is an adapted web-based version of the well-validated measure of processing speed [28]. Performance on the task was correlated with its MCCB equivalent (*r*=0.73) and “g” (*r*=0.74), had the strongest association with functional outcome, a high factor loading (0.81) and is easily translatable.

#### Task 2: Backward Digit Span

This task is a web-based version of the well-validated measure of working memory [29]. Performance on the task was correlated with its MCCB equivalent (*r*=0.34), was strongly associated with functional outcome, and had a short administration time (3 minutes).

#### Task 3: Vocabulary

Participants are shown a target word and asked to select which of 4 words is closest in meaning to the target word [28]. This task was included as a measure of crystallized intelligence based on its correlation with the National Adult Reading Test (*r*=0.64) [30]. Performance on the task did not load onto the web-based “g” in the 9-task factor analysis but was correlated with MCCB “g” (*r*=0.36), associated with functioning, and was the only well-tolerated verbal task.

#### Task 4: Morphed Emotion Identification (Optional Task)

Participants are presented with a face and must decide whether the face looks angry, fearful, happy, or disgusted [31,32]. Faces are morphed between a neutral face and each emotion at varying intensities. The correlation between this task and its MCCB equivalent was low (*r*=0.26), likely reflecting the different methodologies of the tasks. However, the task was correlated with “g” (*r*=0.58), strongly associated with functional outcome, and captured social cognition.

#### Task 5: Matrix Reasoning (Optional Task)

This task is based on the well-validated matrix reasoning test used in the Wechsler Abbreviated Scale of Intelligence II [28,33]. This task was correlated with both its MCCB equivalent (*r*=0.53) and “g” (*r*=0.59), was associated with functional outcome, and had a high factor loading (0.56). However, it was included as an optional task due to its long administration time (up to 15 minutes if all trials are completed).

#### Excluded Tasks

Hartshorne visual working memory and balloon analogue risk task were excluded due to low correlations with “g” (0.3 and 0.11 respectively). Verbal Paired Associates was poorly tolerated by participants, who voted it the “worst task” in their feedback and could not be easily translated. Multiple object tracking met all inclusion criteria, but participants reported difficulties completing it on smaller touchscreen devices, which could not be easily resolved.

#### Validity of CONCA-Derived “g”

We calculated correlations to compare MCCB “g” with 3 measures of “g” from the web-based batteries: (1) original 9-task battery, (2) CONCA 5-task battery, and (3) CONCA 3-task battery. Correlations were similar between MCCB “g” and “g” from all 3 versions (original 9-task battery: *r*=0.78, 95% CI 0.66-0.86; CONCA 5-task battery: *r*=0.78, 95% CI 0.67-0.86; CONCA 3-task core battery: *r*=0.71, 95% CI 0.57-0.81). Finally, the factor analysis was repeated, including only the final selection of CONCA tasks, and indicated that all tasks contributed to “g” with factor loadings between 0.51 and 0.66 (see Table S2 in Multimedia Appendix 1 for full results).

### Part 2: Assessing Cognition in HealthWise Wales

#### Completion Rates

A total of 3889 participants from HealthWise Wales consented to the study (response rate=3889/29,492, 13.19%). Of these, 3679 participants completed at least one cognitive task (3679/3889, 94.6%). Completion of the core battery was high (3135/3889, 80.61%), including 2048 who completed the core battery and both optional tasks (2048/3889, 52.66%; Table 2). After false discovery rate correction, participants with higher scores on the core tasks were more likely to complete at least one optional task (odds ratio 1.4, 95% CI 1.26-1.55; *P*<.001). None of the other variables significantly predicted the completion of the optional tasks (see Table 3).

**Table 2:** Task completion rates and summary statistics.

Task	Scoring	n	Mean (SD)	Median (IQR)
Digit symbol coding	Correct responses in 90 seconds	3679	41.71 (10.72)	41 (34-49)
Backwards digit span	Longest correctly recalled digit span	3199	4.44 (1.62)	4 (3-5)
Vocabulary	Correct responses (Maximum=20)	3135	16.77 (3.17)	17 (15-19)
Emotion identification	Correct responses (Maximum=60)	2319	34.92 (6.54)	35 (30-40)
Matrix reasoning	Correct responses (Maximum=35)	2444	24.08 (5.74)	25 (21-28)

**Table 3:** Predictors of optional task completion. Results of a logistic regression where the outcome is the completion of at least one optional task (1=completed, 0=not completed).

		OR^a^ (95% CI)	*P* value
Core “g”		1.4 (1.26-1.55)	<.001
Age		1.01 (1-1.02)	.14
Gender (reference: women)		0.95 (0.74-1.23)	.79
**Education (reference: none)**			
	GCSE^b^ or O-levels	0.75 (0.45-1.23)	.44
	A-levels	0.76 (0.46-1.22)	.44
	Degree	0.79 (0.48-1.27)	.49
	Postgraduate degree	0.74 (0.44-1.21)	.44
**Device (reference: desktop** **or** **laptop)**			
	Smartphone	0.95 (0.71-1.27)	.79
	Tablet	1.03 (0.77-1.38)	.84
Ever diagnosed with or treated for a mental health problem (reference: none)		1.38 (1.07-1.76)	.07

^a^OR: odds ratio.

^b^GCSE: General Certificate of Secondary Education

#### Sample Characteristics

Sample characteristics were examined, including all participants who had completed at least one cognitive task (N=3679, see Table 4). Most participants were women (2551/3668, 69.55%) and had a mean age of 55.86 (SD 15.05, range 16-93) years. Participants reported high levels of education: 1095 of 3557 (30.78%) reported an undergraduate degree as their highest level of education, and 732 of 3557 (20.58%) reported a postgraduate degree as their highest level of education. Just under half of participants used a laptop or desktop computer to complete the study (1781/3672, 48.5%), while 803 of 3672 (21.87%) used a tablet device, and 1088 of 3672 (29.63%) used a smartphone. The number of participants who reported a previous diagnosis of or treatment for a mental health condition was 1212 of 3309 (36.63%).

**Table 4:** Sample characteristics. Information on population data was obtained from sources dated as close to the point of the Cardiff Online Cognitive Assessment data collection as possible (January 2020).

Sample characteristics			Available data	HealthWise Wales: whole sample, %^a^	Population data for Wales, %
Gender (women; N=3668)			2551 (69.55)^b^	72	50.69^c^
**Highest education level (N=3557)**				—^d^	
		No GCSEs^e^	259 (7.28)^b^		7.3^f^
		GCSE or equivalent	524 (14.73)^b^		30.3^f^
		A-level or equivalent	947 (26.62)^b^		21.3^f^
		Undergraduate degree	1095 (30.78)^b^		29.2^f^
		Postgraduate degree	732 (20.58)^b^		11.9^f^
**Device used (N=3672)**				—	—
	Laptop or desktop		1781 (48.5)^b^		
	Tablet		803 (21.87)^b^		
	Smartphone		1088 (29.63)^b^		
Ever diagnosed with or treated for a mental health problem (N=3309)			1212 (36.63)^b^	32	11^g^
45 years or older (N=3679)			2802 (76.16)^b^	60	47.25^c^
Age (N=3679), median (IQR)			59 (46-67)^g^	—	42.4^h^
WHODAS^i^ Total (N=1033), median (IQR)			5 (1-12)^g^	—	—
HADS^j^ Anxiety (N=1034), median (IQR)			6 (3-10)^g^	—	—
HADS Depression (N=1034), median (IQR)			5 (2-9)^g^	—	—

^a^Published data from HealthWise Wales [16].

^b^Number and percentage values.

^c^Office for National Statistics’ national-level population estimates for Wales in 2020 (note: sex, not gender, was recorded) [34].

^d^Not available.

^e^GCSE: General Certificate of Secondary Education.

^f^Office for National Statistics’ highest qualification data in 2020 [35] (note: these education categories have been mapped as closely as possible to the study data).

^f^Median (IQR) values.

^g^National Survey for Wales 2019-2020 [36].

^h^Office for National Statistics population estimates for the UK and its constituent countries in 2020 [34].

^i^WHODAS: World Health Organization Disability Assessment Schedule.

^j^HADS: Hospital Anxiety and Depression Scale.

#### Cognitive Performance and Demographic Variables

**Figure 1:**
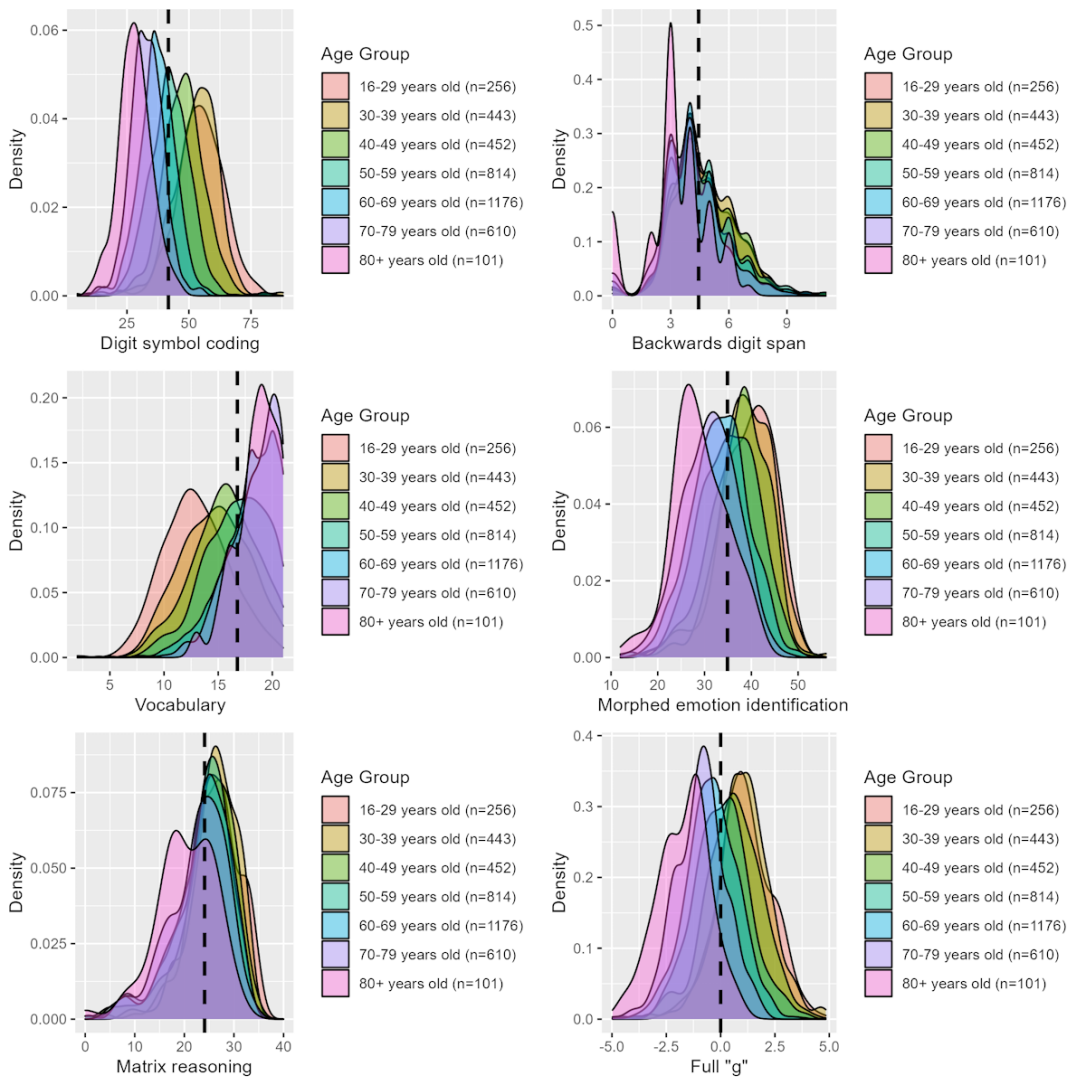
Distributions of performance on tasks by age group. From top left to bottom right, density plots stratified by age group for digit symbol coding, backwards digit span, vocabulary, morphed emotion identification, matrix reasoning, and “g.” The dashed line indicates the mean performance for each task.

There was evidence of a ceiling effect on vocabulary among those aged 60 years and older, as 13.3% (251/1887 participants) of them achieved the maximum score (see Figure 1). Summary statistics for each of the tasks are presented by gender and age group in Table S3 and by educational attainment in Table S4 in Multimedia Appendix 1. These summary statistics can be used to generate age- and gender-adjusted *z* scores using the formula:

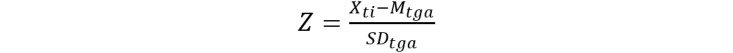



Where *X_ti_* is the score for individual *i* on test *t*, and *M_tga_* and *SD_tga_* represent the mean and SD for test *t* for that individual’s corresponding age group *a* and gender *g*.

Cognitive performance (Full “g”) was associated with age (B=–0.05, SE 0.002; *P*<.001), device (tablet computer: B=–0.27, SE 0.06; *P*<.001; smartphone: B=–0.45, SE 0.05; *P*<.001), and education (degree: B=1.68, SE 0.14; *P*<.001; see Table 5), such that older age, use of a tablet computer or smartphone rather than a laptop or desktop, and lower educational attainment were associated with lower cognitive performance (results for individual tasks can be found in Table S5 in Multimedia Appendix 1). Gender was not associated with “g” (B=–0.002, SE 0.05; *P*=.97) but was associated with performance on 3 tasks: men performed better on vocabulary (B=0.1, SE 0.03; *P*=.004); and matrix reasoning (B=0.2, SE 0.04; *P*<.001), while women performed better on morphed emotion identification (B=–0.24, SE 0.05; *P*<.001). The proportion of variance in full “g” and core “g” explained by demographic variables were 0.34 and 0.36, respectively (adjusted *R*^2^).

**Table 5:** Associations between demographic variables and cognitive performance.

				B^a^		SE		*P* value
**Full “g”**								
	Age			–0.05		0.002		<.001
	Gender (reference: women)			–0.002		0.05		.97
	**Education (reference: no qualifications)**							<.001
		GCSE^b^ or equivalent	1.15		0.14			
		A-levels or equivalent	1.39		0.14			
		Undergraduate degree	1.68		0.14			
		Postgraduate degree	1.87		0.14			
	**Device (reference: desktop or laptop)**							<.001
		Smartphone	–0.45		0.05			
		Tablet	–0.27		0.06			
**Core “g”**								
	Age			–0.04		0.001		<.001
	Gender (reference: women)			–0.02		0.04		.69
	**Education (reference: no qualifications)**							<.001
		GCSE or equivalent	0.64		0.11			
		A-levels or equivalent	0.72		0.11			
		Undergraduate degree	0.88		0.11			
		Postgraduate degree	1.03		0.11			
	**Device (reference: desktop or laptop)**							
		Smartphone	–0.18		0.04		<.001	
		Tablet	–0.13		0.04		.003	

^a^Linear regression coefficients.

^b^GCSE: General Certificate of Secondary Education.

#### Cognitive Performance and Mental Health

Lower scores on the HADS depression subscale were associated with higher general cognitive ability “g” (Full “g”: B=–0.04, SE 0.01; *P*<.001; Core “g”: B=–0.03, SE 0.01; *P*<.001). Lower scores on the HADS anxiety subscale were also associated with higher “g” scores (Full “g”: B=–0.04, SE 0.01; *P*<.001; Core “g”: B=–0.03, SE 0.01; *P*<.001). Self-report of any mental health problem was associated with lower performance on the core CONCA tasks (Core “g”: B=–0.11, SE 0.04; *P*=.01) but this association was not found for Full “g” (B=–0.09, SE 0.05; *P*=.07).

#### Technical Issues

Technical issues were reported by 52 participants (52/3679, 1.4%), and 17 unique problems were identified. A total of 3 of these problems were determined as bugs in the website’s coding and were resolved. Where the problems were the result of bugs in the assessment and participants were unable to view the stimuli, they were given the opportunity to complete the task once the issue was resolved. A total of 5 issues were identified as being specific to those users’ devices, and further technical support was provided by our team to support each participant in completing the tasks if possible. For the remaining 9 issues, insufficient information was provided, and attempts to contact the participants for further information were unsuccessful.

## Discussion

### Principal Findings

The aims of this study were to further develop CONCA to provide a brief measure of “g,” to recruit from a large web-based population study, and to demonstrate CONCA’s application in a health population sample. Results from each aim are outlined in the sections below.

### Part 1: Establishing CONCA

The number of tasks in CONCA was reduced from 9 to 3 core tasks and 2 optional tasks. All these tasks loaded onto a single factor, “g,” which supported our decision to reduce the number of tasks in the battery for the purpose of creating a brief assessment that provides a measure of “g.” The measure of “g” obtained using the tasks from the core CONCA battery was correlated with “g” derived from the MCCB, which indicates that the 3 tasks are sufficient to obtain a valid measure of “g.” This correlation increased when the 2 optional tasks were included, suggesting that while the optional tasks are not essential to derive a measure of “g,” they do have added value.

### Part 2: Assessing Cognition in HealthWise Wales

To demonstrate CONCA’s application in a health population sample, we examined completion rates, technical issues, and performance distributions. This enabled us to determine whether the tasks were sufficiently engaging and challenging for a general population sample. Completion rates for the core CONCA tasks were high, indicating acceptable levels of tolerability and engagement. These rates were similar to those reported in our previous study [14]. Over half the sample completed both additional optional tasks (2048/3679, 52.66% participants), which suggests that participants were sufficiently engaged with the core tasks and our research to be motivated to complete additional measures. It should be highlighted that participants with higher scores on the core tasks were more likely to complete the optional tasks. This suggests that those who find the tasks more difficult may be demotivated and choose not to complete the optional tasks, leading to a less representative sample for these tasks. The number of technical issues reported was low, with only 52 (1.41%) of 3679 participants reporting a problem. Combined with the high completion rates, this suggests that most participants were able to complete the tasks without a problem. The distributions of scores for most of the tasks were relatively normal, except for vocabulary, where there was evidence of a potential ceiling effect, particularly among older participants. This ceiling effect among older people has been identified in a previous report examining the psychometric properties of vocabulary [28].

The relationship between performance on the tasks and age, gender, and education was in the expected direction. Older age and lower education levels were associated with lower scores on all tasks and measures of “g,” except for vocabulary, where older participants performed better. Men performed better on vocabulary and matrix reasoning than women, while women had higher scores on morphed emotion identification. This is consistent with previous studies assessing emotion recognition [37,38] and matrix reasoning [28]. In contrast, a previous report assessing the psychometric properties of the vocabulary task showed marginally better performance in women [28].

We found lower performance among those using touchscreen devices (tablet computer or smartphone) compared to those using a laptop or desktop computer, which is consistent with 2 other studies using these tasks [28,39]. This effect was seen across all the tasks, suggesting that it cannot be explained by response times alone, as some tasks, such as vocabulary, do not have a timed component. The lower performance may be partly explained by screen size, particularly as lower performance was found among participants using smartphones compared to those using tablet computers. This is supported by the findings of Passell et al [39] who demonstrated that performance on digit symbol coding and vocabulary was impacted by screen size, input type, and the type of internet browser used. Device use has been associated with age, gender, and education [39], all of which were controlled for in this study, but there may be other factors that were not measured in this study. Smartphones and tablet computers may be cheaper and more accessible, as they do not rely on a home broadband connection and have relatively straightforward interfaces compared to traditional computers. Therefore, their use may be influenced by socioeconomic factors or computer literacy, which may also be associated with performance on the tasks. Consistent with this, a report by the United Kingdom’s communications regulator, Ofcom, found that people in manual occupations, unemployed, or considered financially vulnerable were most likely to use a smartphone exclusively to access the internet [40]. The portable nature of touchscreen devices means that participants may be more likely to complete the tasks in locations outside the home or while conducting other activities and therefore may be subject to more distractions. These results highlight the importance of controlling for device effects when analyzing cognitive data from web-based studies.

CONCA was designed to be a measure of cognition in psychiatric populations. Therefore, we evaluated whether the mental health measures collected were associated with performance on CONCA. We found that higher levels of depression and anxiety symptoms and a self-reported history of diagnosis or treatment for a mental health problem were associated with lower overall performance on the core CONCA tasks. This suggests that CONCA is sufficiently sensitive to the cognitive differences associated with mental health disorders. This is also a novel finding of the study, as to our knowledge, few studies have examined the relationship between depression and anxiety symptoms and cognition in a general population sample.

### Sample Representativeness

The response rate of 13.9% (3889/29492) raises the issue of participation bias. There was evidence of bias in the demographic distributions of the CONCA sample. Compared to population estimates for Wales, the sample was older, more educated, and predominantly women. We did not stratify the data by ethnicity, as 99% of participants reported their ethnicity as White (3537/3572), which was a consequence of recruiting from the wider HealthWise Wales sample (98% White). The bias reported in this study is in part a reflection of the original HealthWise Wales sample, which has a higher proportion of women, older people, and White people [16]. However, even among the least represented groups (eg, men aged 16-40 years), the number of participants in our sample exceeds the amount of normative data collected for other mental health cognitive batteries, such as the Brief Assessment for Cognition in Schizophrenia [41] and the MCCB [42]. While the sample did contain a higher number of participants with postgraduate degrees than expected, it is important to note that the representation across the education groups was satisfactory, with at least 200 participants in each group. The proportion of participants reporting no qualifications was also comparable to estimates for the Welsh population, which alleviates concerns that the sample may be underrepresented by those with lower educational attainment. We are currently undertaking targeted recruitment to collect data on younger people, with a particular focus on recruiting more men into the sample.

### Strengths and Limitations

We have collected a large cognitive data set on a population sample that spans a wide range of ages and enabled us to derive age-, gender-, and education-based norm scores for CONCA. However, results should be interpreted with consideration of the potential biases in the sample, as detailed below. CONCA has several advantages over existing assessments (such as the Brief Assessment of Cognition in Schizophrenia [BACS] [43] or the Cambridge Neuropsychological Test Automated Battery [CANTAB] [44]), including a user-friendly website designed with input from patient representatives and health professionals, a large normative data set collected on the internet, and the fact that it can be completed on the participants’ own devices (including touchscreen tablets and smartphones) rather than relying on specific hardware or software that can be required for similar assessments.

Sample representativeness is a clear limitation of this study, as highlighted in the previous section. In addition, participants with high scores in the core tasks were more likely to complete the optional tasks. This needs to be considered when interpreting results using the matrix reasoning and morphed emotion identification tasks and is another source of bias. It should also be noted that the response rate for this study was 13.9% (3889/29492). Recruitment for this study commenced in January 2020 and overlapped with the initial months of the COVID-19 pandemic and UK lockdown. There is evidence that the pandemic negatively impacted research participation, with current research participants less able or willing to participate in ongoing research [45]. The main limitations of CONCA include a lack of verbal or episodic memory tasks and a lack of evidence for its use as a longitudinal assessment, although some data on practice effects have been previously published [28].

### Conclusions

CONCA provides a valid measure of “g,” which can be derived using as few as 3 tasks that take no more than 15 minutes. We have demonstrated that the battery is sufficiently engaging and challenging for use in a general population sample, with the potential exception of vocabulary in older adults. Based on our findings, we recommend that CONCA is suitable for use in general population samples and may be particularly useful for studies of the relationship between cognition and mental health, but caution is advised for the use of vocabulary in older adults (60 years and older) given the potential for ceiling effects. Factors that impacted performance on CONCA included age, gender, education, and type of device, and these should be controlled for in analyses as appropriate. The primary purpose of this study was to introduce the new CONCA battery, provide normative data, and demonstrate the associations between CONCA and demographic variables. The recruitment of a web-based normative sample is an important step forward in the development of CONCA, although more work is needed to ensure the data are representative of the population, particularly in terms of education levels. However, we have also reported some novel findings, namely that symptoms of depression and anxiety are associated with cognitive function in a general population sample, as well as demonstrating the effect of device when measuring cognition. Now that we have established normative performance on CONCA, we intend to investigate the clinical use of CONCA, including the development of new features to support health professionals in interpreting their patients’ performance on the battery when administered in a clinical setting.

## Appendix

Multimedia Appendix 1 Supplementary methods and results.

## Abbreviations

BACS: Brief Assessment of Cognition in Schizophrenia
CANTAB: Cambridge Neuropsychological Test Automated Battery
CoMPaSS: Cognition in Mood, Psychosis and Schizophrenia Study
CONCA: Cardiff Online Cognitive Assessment
HADS: Hospital Anxiety and Depression Scale
MATRICS: Measurement and Treatment Research to Improve Cognition in Schizophrenia
MCCB: MATRICS Consensus Cognitive Battery
NCMH: National Centre for Mental Health
SAPPHIRe: Secure Access Portal and Protected HealthWise Wales Information Repository
UKSeRP: UK Secure e-Research Platform
WHODAS: World Health Organization Disability Assessment Schedule

## Data Availability

The data sets generated and analyzed during this study are available through HealthWise Wales on reasonable request [46].
