# Supplementary material for: Cardiff Online Cognitive Assessment in a National Sample: Cross-Sectional Web-Based Study
Source: J Med Internet Res. 2023 Sep 13;25:e46675. doi: 10.2196/46675 (PMC10534289; doi:10.2196/46675)
Supplement: Multimedia Appendix 1 [file jmir_v25i1e46675_app1.docx]

# Multimedia Appendix 1. Supplementary Methods and Results

## Supplementary Table S1: Task Selection Criteria

| **Task** | **Correlation with MCCB equivalent** | **Correlation with MCCB ‘g’** | **Association with functional outcome** | **Factor loading onto online ‘g’** | **Technical issues and participant feedback** | **Easily translate into other languages?** |
| --- | --- | --- | --- | --- | --- | --- |
| **Digit Symbol Coding** | .73 | .74 | B=-3.08, SE=0.47, *P*<.001 | .81 | Issue with response to keyboards (resolved in latest version) | Yes |
| **Morphed Emotion Identification** | .26 | .58 | B=-1.78, SE=0.53, *P*<.001 | .56 |  | Yes |
| **Verbal Paired Associates** | .41 | .44 | B=-1.72, SE=0.5, *P*<.001 | .4 | Voted “worst task” in participant feedback | No |
| **Backward Digit Span** | .34 | .42 | B=-2.03, SE=0.51, *P*<.001 | .43 |  | Yes |
| **Hartshorne Visual Working Memory** | .12 | .30 | B=-1.73, SE=0.51, *P*=0.008 | .66 |  | Yes |
| **Matrix Reasoning Test** | .53 | .59 | B=-1.54, SE=0.43, *P*<.001 | .56 |  | Yes |
| **Balloon Analogue Risk Task** | No equivalent | .11 | B=-1.29, SE=0.39, *P*<.001 | .18 |  | Yes |
| **Multiple Object Tracking** | .34 | .53 | B=-1.92, SE=0.43, *P*<.001 | .7 | Voted “best task” in participant feedback but participants reported difficulties responding on small touchscreen devices | Yes |
| **Vocabulary** | .64 | .36 | B=-1.51, SE=0.51, *P*=0.003 | -.07 |  | No |

## Supplementary Table S2: Factor Analysis (Final CONCA Battery)

All analyses used age and gender corrected scores.

CONCA Core Battery: Examination of the scree plot and parallel analysis indicated 1 factor with an eigenvalue above 1.

CONCA Full Battery: Examination of the scree plot and parallel analysis indicated 1 factor with an eigenvalue above 1.

| **Task** | **CONCA Core Battery: Factor loadings** | **CONCA Full Battery: Factor loadings** |
| --- | --- | --- |
| Digit Symbol Coding | 0.56 | 0.57 |
| Backward Digit Span | 0.56 | 0.52 |
| Vocabulary | 0.51 | 0.55 |
| Morphed Emotion Identification |  | 0.51 |
| Matrix Reasoning |  | 0.66 |

## Supplementary Table S3: Cognitive scores by gender and age group

|  | **Female** | | | | **Male** | | | |
| --- | --- | --- | --- | --- | --- | --- | --- | --- |
|  | N | Mean | SD | Z | N | Mean | SD | Z |
| **Digit Symbol Coding** | |  |  |  |  |  |  |  |
| 16-29 | 217 | 54.32 | 9.6 | 1.18 | 27 | 56.89 | 9.29 | 1.42 |
| 30-39 | 355 | 52.95 | 8.15 | 1.05 | 66 | 52.76 | 11.57 | 1.03 |
| 40-49 | 362 | 47.77 | 8.31 | 0.56 | 72 | 48.11 | 7.94 | 0.6 |
| 50-59 | 591 | 42.58 | 8.7 | 0.08 | 186 | 41.47 | 8.2 | -0.02 |
| 60-69 | 699 | 37.55 | 7.21 | -0.39 | 423 | 36.78 | 7.26 | -0.46 |
| 70-79 | 297 | 32.99 | 6.31 | -0.81 | 272 | 33.2 | 6.96 | -0.79 |
| 80+ | 32 | 28.44 | 7 | -1.24 | 56 | 27.11 | 6.39 | -1.27 |
| **Backward Digit Span** | | |  |  |  |  |  |  |
| 16-29 | 193 | 4.62 | 1.41 | 0.11 | 26 | 4.92 | 1.38 | 0.3 |
| 30-39 | 310 | 4.74 | 1.46 | 0.18 | 56 | 4.8 | 1.86 | 0.22 |
| 40-49 | 320 | 4.74 | 1.66 | 0.19 | 66 | 4.85 | 1.62 | 0.25 |
| 50-59 | 525 | 4.53 | 1.63 | 0.05 | 157 | 4.56 | 1.75 | 0.07 |
| 60-69 | 599 | 4.33 | 1.6 | -0.07 | 364 | 4.39 | 1.57 | -0.03 |
| 70-79 | 255 | 3.98 | 1.49 | -0.29 | 234 | 4.15 | 1.71 | -0.18 |
| 80+ | 27 | 3.48 | 0.98 | -0.59 | 45 | 3.29 | 1.94 | -0.71 |
| **Vocabulary** |  |  |  |  |  |  |  |  |
| 16-29 | 186 | 12.7 | 2.86 | -1.28 | 25 | 15.04 | 3.08 | -0.54 |
| 30-39 | 303 | 14.55 | 3.13 | -0.7 | 52 | 15.54 | 3.27 | -0.39 |
| 40-49 | 316 | 15.38 | 3 | -0.44 | 65 | 16.63 | 2.76 | -0.04 |
| 50-59 | 514 | 16.56 | 2.95 | -0.06 | 155 | 17.12 | 2.75 | 0.11 |
| 60-69 | 593 | 17.92 | 2.58 | 0.36 | 356 | 18.03 | 2.48 | 0.4 |
| 70-79 | 248 | 18.23 | 2.28 | 0.46 | 228 | 18.57 | 2.32 | 0.57 |
| 80+ | 27 | 19 | 1.71 | 0.7 | 45 | 18.36 | 2.06 | 0.5 |
| **Morphed Emotion Identification** | | |  |  |  |  |  |  |
| 16-29 | 139 | 39.55 | 5.82 | 0.71 | 19 | 38.11 | 6.54 | 0.49 |
| 30-39 | 224 | 39.08 | 5.7 | 0.64 | 36 | 37.97 | 5.32 | 0.47 |
| 40-49 | 231 | 37.69 | 5.96 | 0.42 | 46 | 36.63 | 4.95 | 0.26 |
| 50-59 | 392 | 35.57 | 6.13 | 0.1 | 122 | 34.23 | 6.19 | -0.11 |
| 60-69 | 432 | 33.99 | 6.12 | -0.14 | 268 | 32.29 | 5.6 | -0.4 |
| 70-79 | 171 | 31.94 | 6.02 | -0.46 | 174 | 30.67 | 5.78 | -0.65 |
| 80+ | 17 | 30.53 | 5.34 | -0.67 | 33 | 28.42 | 5.98 | -0.99 |
| **Matrix Reasoning** | |  |  |  |  |  |  |  |
| 16-29 | 143 | 25.92 | 4.97 | 0.32 | 21 | 28.05 | 5.44 | 0.69 |
| 30-39 | 233 | 25.79 | 4.7 | 0.3 | 38 | 27.82 | 4.57 | 0.65 |
| 40-49 | 257 | 24.28 | 5.88 | 0.03 | 49 | 26.78 | 5.38 | 0.47 |
| 50-59 | 426 | 23.52 | 5.63 | -0.1 | 123 | 24.74 | 5.71 | 0.12 |
| 60-69 | 451 | 23.57 | 5.85 | -0.09 | 277 | 24.06 | 5.68 | -0.003 |
| 70-79 | 179 | 21.64 | 6.33 | -0.43 | 174 | 23.9 | 5.08 | -0.03 |
| 80+ | 17 | 21.35 | 5.72 | -0.47 | 41 | 19.9 | 6.07 | -0.73 |

## Supplementary Table S4: Cognitive scores by educational attainment

|  | **N** | **Mean** | **SD** | **Z** |
| --- | --- | --- | --- | --- |
| **Digit Symbol Coding** |  |  |  |  |
| No GCSEs | 258 | 34.67 | 8.36 | -0.66 |
| GCSE or equivalent | 524 | 39.07 | 9.88 | -0.25 |
| A-levels or equivalent | 943 | 41.46 | 10.14 | -0.02 |
| Undergraduate degree | 1092 | 43.35 | 10.91 | 0.15 |
| Post-graduate degree | 728 | 44.52 | 10.81 | 0.26 |
| **Backward Digit Span** |  |  |  |  |
| No GCSEs | 217 | 3.64 | 1.5 | -0.49 |
| GCSE or equivalent | 449 | 4.15 | 1.51 | -0.18 |
| A-levels or equivalent | 802 | 4.34 | 1.57 | -0.06 |
| Undergraduate degree | 956 | 4.63 | 1.57 | 0.12 |
| Post-graduate degree | 662 | 4.8 | 1.7 | 0.22 |
| **Vocabulary** |  |  |  |  |
| No GCSEs | 213 | 14.85 | 3.42 | -0.6 |
| GCSE or equivalent | 437 | 16.14 | 3.12 | -0.2 |
| A-levels or equivalent | 784 | 16.42 | 3.19 | -0.11 |
| Undergraduate degree | 938 | 17.19 | 3.1 | 0.13 |
| Post-graduate degree | 650 | 17.68 | 2.74 | 0.29 |
| **Morphed Emotion Identification** |  |  |  |  |
| No GCSEs | 144 | 30.97 | 6.4 | -0.61 |
| GCSE or equivalent | 321 | 33.43 | 5.96 | -0.23 |
| A-levels or equivalent | 571 | 34.91 | 6.58 | -0.002 |
| Undergraduate degree | 699 | 35.99 | 6.39 | 0.16 |
| Post-graduate degree | 501 | 35.95 | 6.4 | 0.16 |
| **Matrix Reasoning** |  |  |  |  |
| No GCSEs | 163 | 18.37 | 6.98 | -0.99 |
| GCSE or equivalent | 331 | 22.26 | 5.93 | -0.32 |
| A-levels or equivalent | 619 | 24.09 | 5.18 | 0.002 |
| Undergraduate degree | 738 | 25.12 | 5.15 | 0.18 |
| Post-graduate degree | 508 | 25.91 | 4.94 | 0.32 |

## Supplementary Table S5: Associations between demographic variables and cognitive performance

|  | **B** | **SE** | ***P*** |
| --- | --- | --- | --- |
| **Digit Symbol Coding** |  |  |  |
| Age | -0.05 | 0.001 | <.001 |
| Gender (reference: women) | -0.03 | 0.03 | .36 |
| Education (reference: no qualifications) |  |  |  |
| *GCSE / O-levels* | 0.4 | 0.08 | <.001 |
| *A-levels* | 0.48 | 0.08 | <.001 |
| *Degree* | 0.6 | 0.08 | <.001 |
| *Post-graduate degree* | 0.73 | 0.08 | <.001 |
| Device (reference: desktop/laptop) |  |  |  |
| *Smartphone* | -0.13 | 0.03 | <.001 |
| *Tablet* | -0.04 | 0.03 | .2 |
| **Backwards Digit Span** |  |  |  |
| Age | -0.01 | 0.001 | <.001 |
| Gender (reference: women) | -0.002 | 0.04 | .97 |
| Education (reference: no qualifications) |  |  |  |
| *GCSE / O-levels* | 0.56 | 0.12 | <.001 |
| *A-levels* | 0.65 | 0.12 | <.001 |
| *Degree* | 0.81 | 0.12 | <.001 |
| *Post-graduate degree* | 0.91 | 0.12 | <.001 |
| Device (reference: desktop/laptop) |  |  |  |
| *Smartphone* | -0.2 | 0.05 | <.001 |
| *Tablet* | -0.18 | 0.05 | <.001 |
| **Vocabulary** |  |  |  |
| Age | 0.03 | 0.001 | <.001 |
| Gender (reference: women) | 0.1 | 0.03 | .004 |
| Education (reference: no qualifications) |  |  |  |
| *GCSE / O-levels* | 0.52 | 0.1 | <.001 |
| *A-levels* | 0.72 | 0.1 | <.001 |
| *Degree* | 0.99 | 0.09 | <.001 |
| *Post-graduate degree* | 1.11 | 0.1 | <.001 |
| Device (reference: desktop/laptop) |  |  |  |
| *Smartphone* | -0.31 | 0.04 | <.001 |
| *Tablet* | -0.15 | 0.04 | <.001 |

|  | **B** | **SE** | ***P*** |
| --- | --- | --- | --- |
| **Emotion Identification** |  |  |  |
| Age | -0.03 | 0.001 | <.001 |
| Gender (reference: women) | -0.24 | 0.05 | <.001 |
| Education (reference: no qualifications) |  |  |  |
| *GCSE / O-levels* | 0.43 | 0.14 | .002 |
| *A-levels* | 0.56 | 0.14 | <.001 |
| *Degree* | 0.71 | 0.13 | <.001 |
| *Post-graduate degree* | 0.73 | 0.14 | <.001 |
| Device (reference: desktop/laptop) |  |  |  |
| *Smartphone* | -0.2 | 0.05 | <.001 |
| *Tablet* | -0.11 | 0.05 | .03 |
| **Matrix Reasoning** |  |  |  |
| Age | -0.02 | 0.001 | <.001 |
| Gender (reference: women) | 0.2 | 0.04 | <.001 |
| Education (reference: no qualifications) |  |  |  |
| *GCSE / O-levels* | 1.03 | 0.13 | <.001 |
| *A-levels* | 1.31 | 0.12 | <.001 |
| *Degree* | 1.46 | 0.12 | <.001 |
| *Post-graduate degree* | 1.59 | 0.12 | <.001 |
| Device (reference: desktop/laptop) |  |  |  |
| *Smartphone* | -0.49 | 0.05 | <.001 |
| *Tablet* | -0.2 | 0.05 | <.001 |
